# Successful resuscitation for cardiac arrest due to severe accidental hypothermia accompanied by mandibular rigidity: a case of cold stiffening mimicking rigor mortis

**DOI:** 10.1186/s12245-018-0205-8

**Published:** 2018-11-14

**Authors:** Naofumi Bunya, Keigo Sawamoto, Ryuichiro Kakizaki, Kenshiro Wada, Yoichi Katayama, Hirotoshi Mizuno, Hiroyuki Inoue, Shuji Uemura, Keisuke Harada, Eichi Narimatsu

**Affiliations:** Department of Emergency Medicine, Sapporo Medical University, S1W16 Chuo-ku, Sapporo, Hokkaido 060-8543 Japan

**Keywords:** Accidental hypothermia, Extracorporeal membrane oxygenation, Rigor mortis, Potassium

## Abstract

**Background:**

In cases of severe accidental hypothermia, it was recommended that resuscitation should be continued until the patient has rewarmed, as hypothermia itself can preserve cerebral function, and hypothermic cardiac arrest is reversible. During cardiopulmonary resuscitation for normothermic patients, muscle rigidity suggests the initiation of postmortem changes such as rigor mortis and can lead to the termination of resuscitation. Currently, the prognosis of cardiac arrest due to severe accidental hypothermia accompanied by rigidity is unknown.

**Case presentation:**

A 29-year-old woman was found unresponsive near a snowy mountain trail. Upon discovery, she was found to be in cardiac arrest with an initial asystole rhythm and exhibited mandibular rigidity. On admission, her core temperature was 22 °C. Although cardiac arrest continued, and she showed no response to normal resuscitation, blood gas analysis revealed that her initial serum potassium level was 5.4 mmol/L. Extracorporeal membrane oxygenation (ECMO) for systemic perfusion and rewarming was initiated. After ECMO was introduced, return of spontaneous circulation was achieved. She showed no neurological impairments at discharge.

**Conclusions:**

Muscle rigidity does not rule out the possibility of resuscitation in patients with severe accidental hypothermia under cardiac arrest. Serum potassium levels may assist in deciding whether ECMO should be introduced, even if a patient is in asystole. This knowledge may help emergency physicians to save the lives of such patients.

## Background

In cases of severe accidental hypothermia (core temperature ≤ 30 °C), it is recommended that resuscitation should be continued until the patient has rewarmed, because hypothermia itself provides cerebral protection despite a prolonged duration of cardiac arrest [1]. Several studies have demonstrated better outcomes after cardiac arrest due to severe accidental hypothermia in patients receiving extracorporeal membrane oxygenation (ECMO) or cardiopulmonary bypass (CPB) [2, 3].

During cardiopulmonary resuscitation (CPR) for normothermic patients, the appearance of muscle rigidity is often regarded as an irreversible change that signals the initiation of postmortem changes, such as rigor mortis, and the presence of obvious rigor mortis is regarded as a contraindication to starting resuscitation attempts [4]. However, the implications of muscle rigidity on the outcome of cardiac arrest due to accidental hypothermia have not been formally studied. We report a case of cardiac arrest due to severe accidental hypothermia accompanied by mandibular rigidity, in which resuscitation was successfully achieved by ECMO. The serum potassium level was used as part of the decision-making process.

## Case presentation

**Fig. 1:**
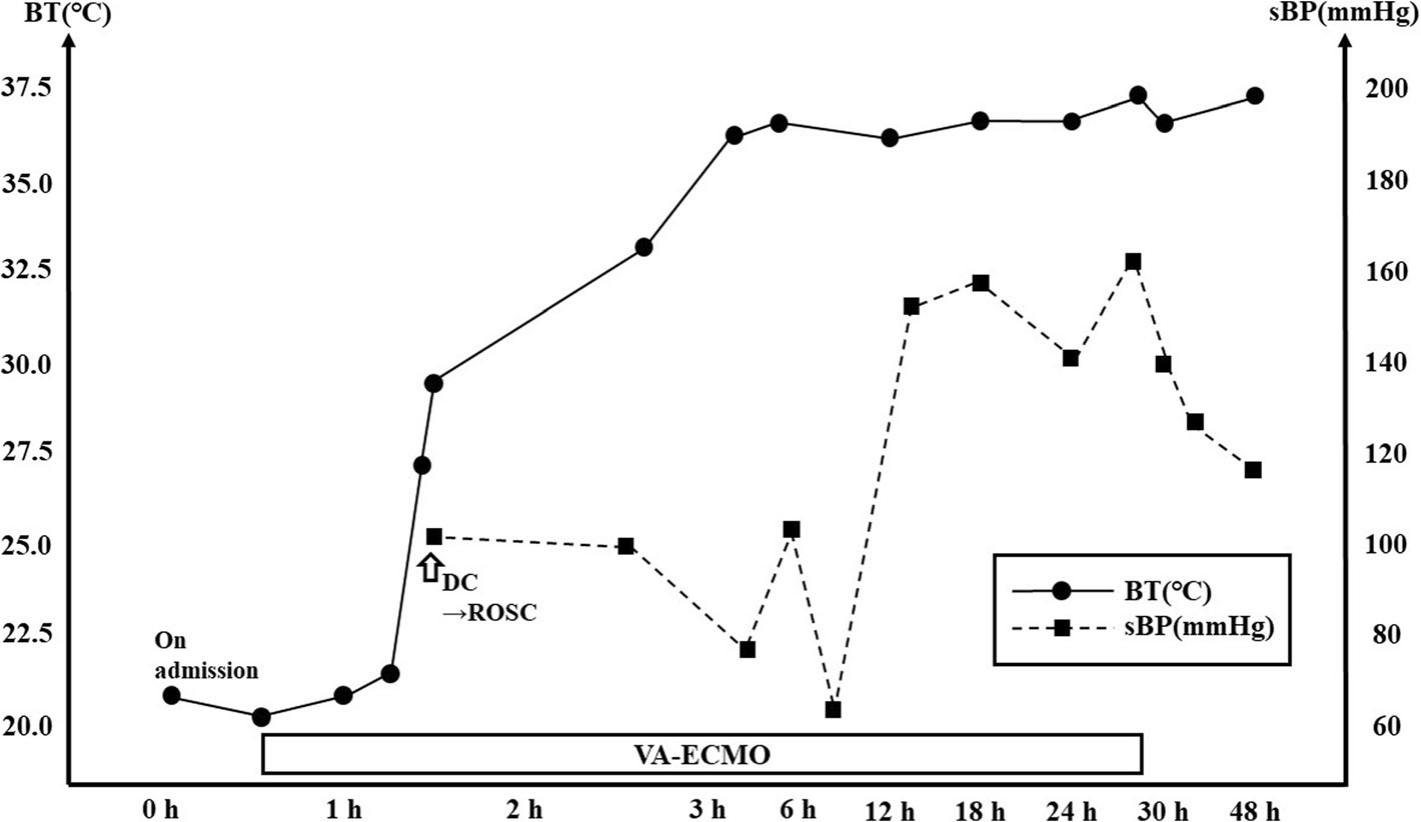
Clinical course

A 29-year-old woman was found unresponsive near a snowy mountain trail in winter. On that day, the outside air temperature ranged from − 2.0 to 1.0 °C. When emergency medical services arrived on the scene, they found that the patient was in cardiac arrest and in asystole. It was noticed that she also had mandibular rigidity. On the way to the hospital, emergency medical service staff were unable to insert an oral airway device for ventilation because the mandibular rigidity prevented sufficient mouth opening. The chest was compressible. CPR was performed with manual chest compression and bag valve mask ventilation ratio of 30:2 during transportation. When she was admitted to the hospital, 52 min had passed since she was discovered. She remained in cardiac arrest with an asystolic cardiac rhythm. Her initial core temperature was 22 °C measured by bladder thermistor. Her mandibular rigidity remained, and neck mobility was also restricted. Both elbows and knees could be passively bent with resistance and the chest wall was not stiff. We were concerned that postmortem changes (i.e., rigor mortis) had begun. We tried to force open her mouth for intubation and found that it could be slightly opened. Although this was insufficient to visualize the vocal cords with a conventional laryngoscope because of the impossibility of controlling it, we were able to insert an “Airway scope™” video laryngoscope, which allowed successful intubation. Although the patient was in cardiac arrest due to severe accidental hypothermia, which indicated an enhanced possibility of successful resuscitation, the mandibular rigidity connected, along with the supposition of rigor mortis initiation made us believe that it would be difficult to resuscitate successfully. However, her initial blood gas analysis revealed that her serum potassium level was 5.4 mmol/L (Table 1). We decided to use veno-arterial ECMO to generate effective systemic perfusion and to rewarm the patient. After ECMO initiated, her temperature transiently dropped to 20.9 °C, then little by little turned to rise. Although she initially remained in asystole after ECMO was introduced, low amplitude ventricular fibrillation gradually appeared as her temperature rose, and the amplitude of ventricular fibrillation progressively increased. At this time, she started breathing spontaneously and her mandibular rigidity disappeared. Once her temperature reached 28 °C, we used defibrillation to achieve sinus rhythm and restore her circulation. Her temperature reached 36 °C after 165 min of ECMO (Fig. 1).

**Table 1:** Laboratory data on admission

Hematology			Biochemistry		Hemostasis		
WBC	15,200 cells/μL		CRP	1.00 mg/dL	PT	13.8 s	
RBC	605 × 10^4^ cells/μL		Procalcitonin	0.55 ng/mL	PT ratio	1.18	
Hb	18.0 g/dL		AST	800 IU/L	PT-INR	1.18	
Ht	58.0%		ALT	277 IU/L	APTT	32.4 s	
Plt	14.7 × 10^4^ /μL		LDH	1552 IU/L	Fibrinogen	289 mg/dL	
			ALP	285 IU/L	FDP	8.2 μg/mL	
Blood gas analysis			T.Bil	0.5 mg/dL	d-dimer	2.9 μg/mL	
pH	6.819		BUN	19 mg/dL	ATIII	92%	
PaCO_2_	85.4 mmHg		Cre	0.60 mg/dL			
PaO_2_	30.0 mmHg		Na	138 mEq/L			
HCO_3_^−^	6.8 mmol/L		K	5.4 mEq/L			
BE	−18.8 mmol/L		Cl	94 mEq/L			
Lactate	11.5 mmol/L		Ca	9.4 mEq/L			
SaO_2_	98.4 %		CPK	1070 IU/L			
K	5.4 mmol/L		Mb	7439 ng/mL			
Na	138 mmol/L		TP	8.3 g/dL			
			Alb	4.7 g/dL			
			Glu	18 mg/dL

After admission to the intensive care unit, her hemodynamic status gradually improved, ECMO was stopped on day 2, and she was discharged from the intensive care unit on day 12. The content of the patient’s bag revealed empty packages of a sleeping drug and a half-finished bottle of alcohol. On regaining consciousness, the patient confessed that she consumed the sleeping drugs and alcohol in an effort to commit suicide.

She was moved to another ward for rehabilitation and showed no neurological impairments on day 42 after the cardiac arrest event.

## Discussion and conclusions

This case highlights two important clinical issues. First, muscle rigidity mimicking rigor mortis does not rule out the possibility of resuscitation in patients who are in cardiac arrest due to severe accidental hypothermia. Second, serum potassium levels may assist in deciding whether ECMO should be introduced.

Muscle rigidity mimicking rigor mortis does not rule out the possibility of resuscitation in patients who are in cardiac arrest due to severe accidental hypothermia. Rigor mortis is a postmortem change that causes muscular stiffening. Although all muscles in the body are affected, it begins with the eyelids, neck, and mandible and then spreads from the upper body towards the legs [5]. Rigor mortis is one of the signs of irreversible death and is an obvious justification for not starting cardiopulmonary resuscitation [4]. In our case, although the patient exhibited mandibular rigidity, other major joints were not rigid. At the initial examination, we considered this mandibular rigidity as a sign of the beginning of rigor mortis. Notably, in severe accidental hypothermia, clinical musculoskeletal manifestations may present as increased muscle tone, shivering, rigidity or pseudo-rigor mortis, paravertebral spasm, and opisthotonos [6]. This pseudo-rigor mortis or cold stiffening must not be mistaken for true rigor mortis [5, 6]. The decision to terminate resuscitation must not be based on muscle rigidity alone in patients with severe accidental hypothermia.

Secondly, serum potassium levels can assist in deciding whether we should proceed to introduce ECMO or consider termination of CPR. There were several reports that high serum potassium levels were associated with poor outcomes [3, 7]. Serum potassium has been used as an indicator of non-survival because it is a marker of hypoxia prior to cooling [3, 8]. Therefore, clinicians may consider termination of CPR in patients with severe accidental hypothermia who are in cardiac arrest if they have high serum potassium levels. Although there is no unified view, some researchers recommend a potassium levels of 10 mmol/L or 12 mmol/L as the cutoff above which CPR is considered to be futile [3, 7, 9, 10]. There was a report that a patient with cardiac arrest due to accidental hypothermia was successfully resuscitated with serum potassium level of 9.0 mmol/L [11]. Hence, when the potassium level is less than 9 mmol/L, cardiopulmonary resuscitation with ECMO should be considered. In our case, although the patient presented with mandibular rigidity and refractory cardiac arrest with asystole rhythm, her serum potassium level was < 9.0 mmol/L and she was successfully resuscitated using ECMO and did not show any neurological abnormalities.

As the prognosis of hypothermic cardiac arrest is affected by multiple factors, serum potassium level is not the only criterion to decide if a victim of hypothermic cardiac arrest can benefit from ECMO. The HOPE score, which predicts the survival rate of patients with hypothermic cardiac arrest, was derived from several studies [12]. The score contains six predictors, including serum potassium, age, gender, core temperature, presence or absence of asphyxia, and CPR duration. The probability of survival in the present case was calculated as 89%. In our case, serum potassium level was used as a deciding factor to introduce ECMO. The HOPE score will work more effectively to save patients in hypothermic cardiac arrest.

In conclusion, in patients with severe accidental hypothermia and cardiac arrest, muscle rigidity does not rule out the possibility of resuscitation, because this muscle rigidity may be due to pseudo-rigor mortis or cold stiffening. Serum potassium levels may assist in deciding whether ECMO should be introduced or termination of CPR should be considered. This knowledge will help emergency physicians in saving the lives of such patients.

## Abbreviations

BT: Body temperature
DC: Direct current
ROSC: Return of spontaneous circulation
sBP: Systolic blood pressure
ECMO: Extracorporeal membrane oxygenation
